# BioSAXS–an emerging method to accelerate, enrich and de-risk antimicrobial drug development

**DOI:** 10.3389/fphar.2022.947005

**Published:** 2022-08-23

**Authors:** Christoph Rumancev, Axel Rosenhahn, Kai Hilpert

**Affiliations:** ^1^ Analytical Chemistry, Biointerfaces, Ruhr-University Bochum, Bochum, Germany; ^2^ Institute of Infection and Immunology, University of London, London, United Kingdom

**Keywords:** Small angle X-ray scattering, BioSAXS, SAXS, drug development, antimicrobials, antibiotics, mode of action, resistance

## Abstract

Antimicrobial resistance is a worldwide threat to modern health care. Low-profit margin and high risk of cross-resistance resulted in a loss of interest in big pharma, contributing to the increasing threat. Strategies to address the problem are starting to emerge. Novel antimicrobial compounds with novel modes of action are especially valued because they have a lower risk of cross-resistance. Up to now determining the mode of action has been very time and resource consuming and will be performed once drug candidates were already progressed in preclinical development. BioSAXS is emerging as a new method to test up to thousands of compounds to classify them into groups based on ultra-structural changes that correlate to their modes of action. First experiments in *E. coli* (gram-negative) have demonstrated that using conventional and experimental antimicrobials a classification of compounds according to their mode of action was possible. Results were backed up by transmission electron microscopy. Further work showed that also gram-positive bacteria (*Staphylococcus aureus*) can be used and the effects of novel antimicrobial peptides on both types of bacteria were studied. Preliminary experiments also show that BioSAXS can be used to classify antifungal drugs, demonstrated on *Candida albicans*. In summary, BioSAXS can accelerate and enrich the discovery of antimicrobial compounds from screening projects with a novel mode of action and hence de-risk the development of urgently needed antimicrobial drugs.

## Introduction

According to the World Health Organization (WHO), SARS-CoV-2 caused over a period of 2 years and 3 months about 6 million deaths. In a new study from 2022, it was reported that in 2019 about 1.27 million people died as a consequence of infections caused by resistant bacteria and 4.95 million deaths were associated with antimicrobial resistance (Murray et al., 2022). These numbers show the dimensions of the burden caused by infectious microorganisms where no treatment is available. Antimicrobial resistance is an ongoing problem that intensified with a loss of investment and interest in most larger pharma companies in the last decades. In addition, the enthusiastic switch from cell-based assays to target-based high throughput screening in the pharma industry resulted in the disappointing failure of this approach (Payne et al., 2007; Boyd et al., 2021). Consequently, a gap in antimicrobial drug development appeared, for nearly 40 years no new structural class was developed. Only in 2000, a novel class was introduced to the market (Boyd et al., 2021). Developing novel antimicrobial drugs are urgently needed, however, the financial incentive to do so is small. Competing antimicrobial drugs are very cheap compared to other drugs, the risk of development of resistance is high, courses of antibiotics are short, the risk of failure from entering Phase I clinical trial to approval is about 81%, and the cost to get a drug candidate all the way to FDA approval and beyond is in the order of 2 billion US dollars (DiMasi et al., 2016; Mullard, 2016). There are novel treatment options developed mainly in academia and small pharma companies and there are national and international efforts underway to address this situation (Czaplewski et al., 2016; Boyd et al., 2021).

**FIGURE 1:**
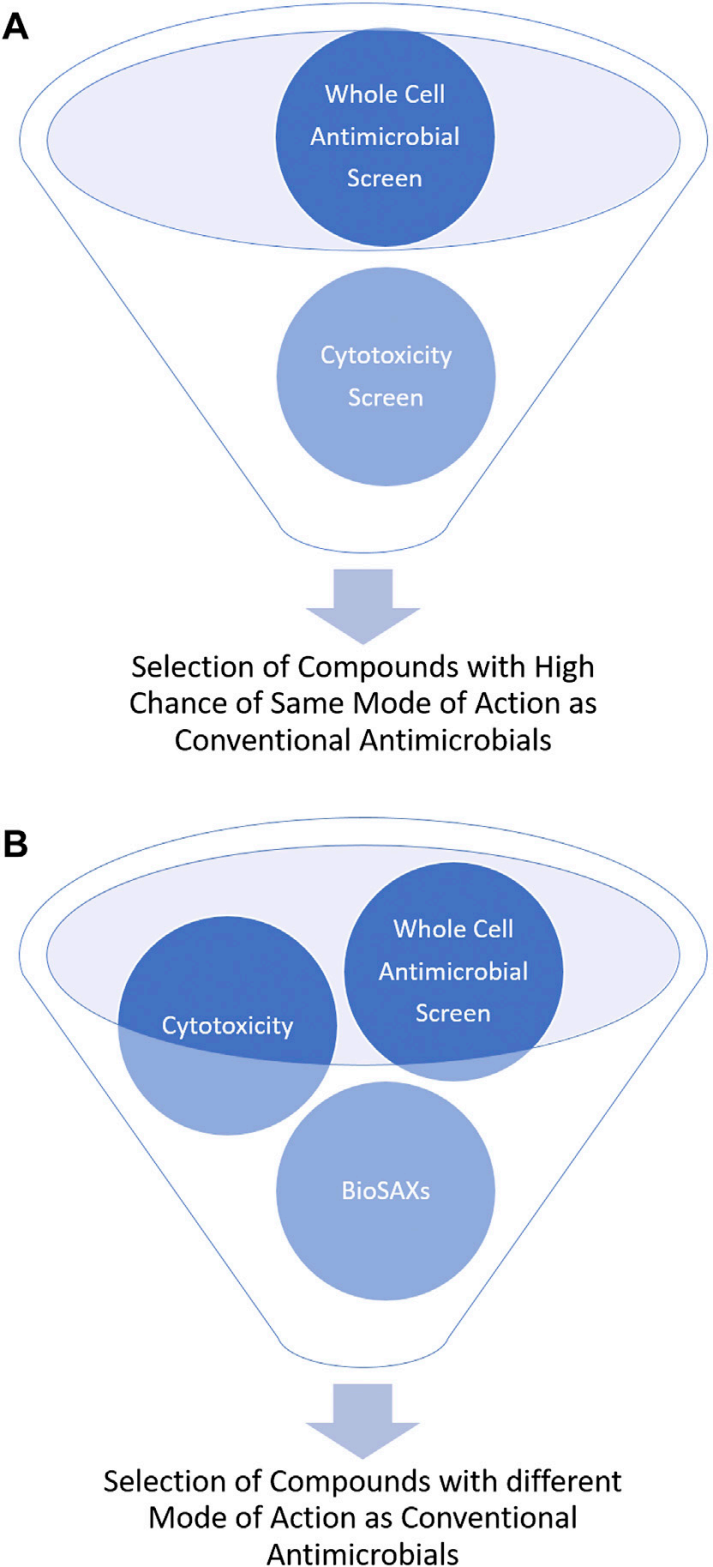
Schematic representation of the outcome of antimicrobial drug development without **(A)** and with BioSAXS **(B)** as an additional screening tool.

Most antibiotics are used for decades and are thus prone for resistance development. Antimicrobial drugs that have a new mode of action are urgently needed. However, determining the mode of action of novel compounds is often not easy and not available in a high-throughput approach. Small-angle x-ray scattering for biological samples (BioSAXS) has in the recent years emerged as promising new approach to classify modes of action of antimicrobial compounds. Here in this mini-review, we describe BioSAXS as a medium-throughput method that has shown the potential to accelerate, enrich and de-risk the antimicrobial drug pipeline. To reduce the risk of cross-resistance and consequently, early failure of the new drug, a novel mode of action/target of the drug candidates is critical. BioSAXS has demonstrated the ability to class antimicrobial compounds according to their modes of action by recording and analyzing ultrastructural changes in the targeted microbes. Since a measurement takes only a few seconds hundreds or up to thousands of compounds can be measured and classed according to their mode of action (Von Gundlach et al., 2016a). Once compared to conventional antibiotics, compounds with novel modes of action can be identified and selected for further drug development. This procedure can therefore enrich compounds with novel modes of action and also de-risk the chance of cross-resistance with conventional antibiotics, see Figure 1.

### The principle of small-angle x-ray scattering

**FIGURE 2:**
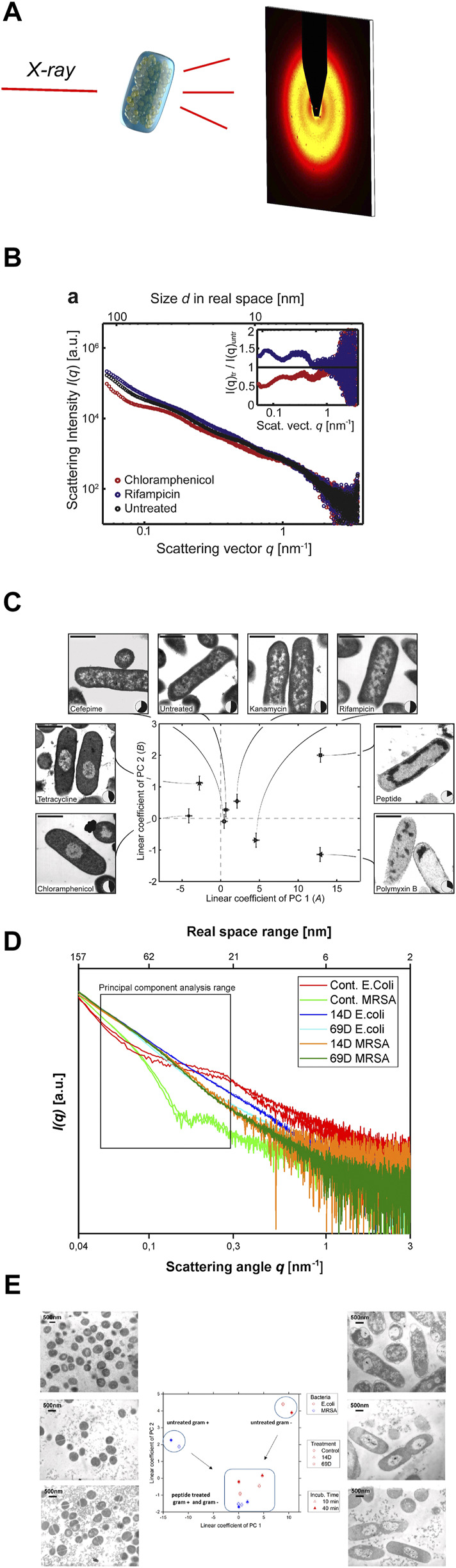
**(A)** Schematic representation of the experimental setting to screen antimicrobial agent-induced ultrastructural intracellular changes by SAXS**. (B)** [Reprint Permission from (Von Gundlach et al., 2016a)]Scattering curves of untreated *E. coli* cells (black), as well as *E. coli* cells after treatment with chloramphenicol (red) and rifampicin (blue). **(C)** [Reprint Permission from (Von Gundlach et al., 2016a)]Result of the Principal Component Analysis (PCA) of *E. coli* cells, untreated and treated with various antimicrobial compounds. Replicate measurements were performed and the error bars denote the standard deviation. Treated and untreated *E. coli* were also imaged by electron microscopy to correlate the shifts in the SAXS data with real space structural information. The nucleoid solidity was also measured by image analysis of 100–300 cells. The scale shows 1 bar of 1 µm. Taken from (von Gundlach et al., 2019) **(D)** Scattering curves from *E. coli* and MRSA treated with AMPs 14D and 69D measured at the P12 BioSAXS beamline at PETRA III (Hamburg, Germany), taken from (von Gundlach et al., 2019) **(E)** PCA graph of two antimicrobial peptides (14D and 69D) that were used to treat *E. coli* and MRSA for 10 min and 40 min. Transmission electron microscopy (TEM) pictures of MRSA (left) and *E. coli* (right). The top pictures are untreated, the middle section was treated with 14D and the base picture was treated with 69D. Taken from (von Gundlach et al., 2019).

Small angle X-ray scattering (SAXS) is a powerful tool to study macromolecules and nanomaterials in solution or suspension (Blanchet et al., 2015), (Svergun and Koch, 2003), (Blanchet and Svergun, 2013). There are existing also studies with SAXS on peptides and macromolecules that can be used for the treatment of infections (Angelova et al., 2019; Feger et al., 2020; Zhang et al., 2021). The unique analytical possibilities are a result of the combination of short wavelength and a large penetration depth of the used photons which allows to investigate large volumes that contain millions of objects in less than a second (Franke and Svergun, 2020). The short wavelength of the X-rays leads to a high sensitivity of diffraction techniques for small structures down to the atomic level. SAXS provides nanoscale structural information on disordered systems and due to the large penetration depth is capable to resolve inner structures of objects. The elastic scattering of the X-rays is sensitive to locally inhomogeneous objects that are composed of domains with differing scattering densities. In most cases the excess scattering density determines the contrast e.g., of particles in different liquids or materials containing different phases or domains (Feigin and Svergun, 1987). After the sample has been irradiated with X-rays, the scattered photons are recorded with a spatially resolving 2D detector (Figure 2A). As no lenses are used, the detector measures the Fourier-transform of the objects, albeit only the intensity can be recorded and the information about the phase of the radiation is lost. Thus, it is impossible to obtain images of the investigated objects by direct inversion.

While the great benefit of SAXS to retrieve structural information averaged across thousands of particles in the solution provides an excellent statistic over cellular ensembles, it comes with the penalty that the extraction of the structural information from the measured data is challenging due to the phase problem, similar to X-ray crystallography. To extract structural information about the sample, suitable models have to be assumed and adapted to match the recorded data. Typical experiments for SAXS are the elucidation of protein structures in solution (Zhang et al., 2007; Dirkmann et al., 2018; Hura et al., 2019), macromolecules in solution (Brosey and Tainer, 2019; Franke and Svergun, 2020) and *in situ* SAXS analysis of growth of silver nanoparticles (Garcia et al., 2020), *in situ* SAXS analysis of growth kinetics of gold nanoparticles (Chen et al., 2021)in solutions. While it is also possible to measure more complicated hetereogeneous systems with SAXS, the interpretation of the data becomes increasingly difficult.

Another example is the use of SAXS to locally analyze the organization of biological systems like intracellular and intraorganelle structures, which is particularly challenging due to the presence of mainly disordered structures across a large variety of lengthscales. The internal structure of melanosomes extracted from wild type and glaucoma developing double congenic knock-out mice were compared by an anaylsis of the Porod decays and it was found that the genetic modification caused a porous internal structure of the organelles that could be responsible for the progression of the disease (Gorniak et al., 2014). Scanning X-ray diffraction measurements can reveal information on nanoscale in whole living eukaryotic cells. in a X-ray diffraction study structural information of living and chemically fixed cells was compared. Structural differences could be observed on a scale between 30 and 50 nm. (Weinhausen et al., 2014). Beyond wild type cells, SAXS was used to study the structure of huntingtin amyloid aggregates expressed in HeLa cells. Oligomeric structures were detected which were not visible in the light microscope, and the structural information of the repeating units in the inclusion bodies was obtained. (Rumancev et al., 2021). The cells in this experiment were cryogenically fixed and could be studied in their natural state, without fixation or staining.

Due to its ability to sensitively probe structural alterations, SAXS was early on applied in antimicrobial research, initially with a focus on antimicrobial agents that target the cell walls. One of the first applications of SAXS in solution to investigate antimicrobial mechanisms of action was on human LL-37 peptide which can cause membrane damage. A similar effect was observed with melittin and the frog skin peptide PGLa. The effect of LL-37 was studied with SAXS on dipalmitoyl-phosphatidylcholine lipid model. Both the change in size and structure after application of LL-37 was visible in SAXS measurements (Sevcsik et al., 2007). In another study, SAXS in solution was used to investigate the effect of antimicrobial peptides on the lipid membrane. Natural peptides such as cecropin A, LL-37, indolicidin, aurein 1.2, magainin II were studied (Nielsen et al., 2021). Here a change in lipid composition associated with increased lipid transport that could lead to apoptosis was observed.

The power of SAXS results from the high statistics of information on the nanoscale obtained in single measurements which is possible due to the large penetration depth of the X-rays. Structural information from millions of cells can be obtained in an averaged way in single measurements. This advantage can be used for studying cellular responses on the nanoscale, such as the response of cells or bacteria to antimicrobial compounds. First experiments on antibiotic mechanisms of action were studied with BioSAXS experiments (EMBL, Hamburg) at P12 Beamline (Petra III, DESY), and the information from the BioSAXS curves was compared with PCA to determine differences between multiple mechanisms of action (von Gundlach et al., 2016a; Von Gundlach et al., 2016b; Hilpert et al., 2021). Below we describe the results obtained from the first experiments using BioSAXS to classify modes of action of antimicrobial compounds on microorganisms.

### Proof of principle

The first proof of principle experiment was performed on *Escherichia coli* cells and a selection of standard antibiotics plus a novel antimicrobial peptide. The antibiotics chloramphenicol, tetracycline, cefepime, kanamycin, rifampicin and polymyxin B were investigated (Von Gundlach et al., 2016a). In the first experiment, the bacteria were treated with antibiotics and a peptide for 4 h and then fixed in glutaraldehyde. To obtain enough scattering data the experiments were performed at a bacteria concentration of 1 × 10^8^ CFU/ml. The samples were examined at the P12 beamline at the European Molecular Biology Laboratory (EMBL) in Hamburg. Here the advantage of the high intensity of the synchrotron beam can be used, which translates into only 1 s per measurement, see Figure 2B. The internal bacterial structure with high polydispersity is difficult to describe with the usual scattering models. A new analysis method had to be developed. A principal component analysis (PCA) was used to determine the differences in the SAXS curves. PCA makes it possible to extract and represent the key differences from several hundred SAXS curves. Ultrastructural changes occur based on the effects of the antimicrobial compound but also on the bacterial response to that threat. The PCA could indeed be used to analyse the SAXS curves and by plotting the coefficients of the first two principal components. Differences could be visualized, which were difficult to see in the original SAXS curves, see Figure 2B. As a control experiment, we performed transmission electron microscopy (TEM) on *E. coli* cells with the same antibiotics including the peptide, see Figure 2C. Whereas tetracycline and chloramphenicol show very similar TEM images, they could successfully be distinguished by using BioSAXS and a PCA analysis. The only antibiotic that could not be clearly distinguished by BioSAXS and TEM from untreated bacteria was cefepime. The novel antimicrobial peptide with an unknown mode of action could be distinguished from other antibiotic classes. This shows the potential of the method to classify novel compounds according to the mode of action and select candidates that differ in their ultrastructural changes from conventional antibiotics. In further experiments, time series (5, 30, 60 and 240 min) on *E. coli* and selected antibiotics were performed and it was shown that ultrastructural changes can be observed over time and as expected change over time. It was also possible to discriminate between bacteriostatic and bactericidal activities.

Furthermore, in another experiment (Hilpert et al., 2021), the effect of designed hybrid peptides on the structure of *E. coli* was investigated. Two different classes of antimicrobial peptides, proline-rich peptides and arginine-isoleucine-rich peptides were combined to enhance the antimicrobial activity of the hybrid peptides. The proline-rich peptides act on ribosomes whereas the arginine-isoleucine-rich peptides have an unknown mode of action. These new hybrid peptides showed up to 6 times stronger antimicrobial activity and showed different ultrastructural changes in the BioSAXS measurement than the parent peptides. PCA clearly distinguished the two parent peptides from each other, confirming the initial hypothesis of two different mechanisms of action. One of the two hybrid peptides showed a clear deviation in PCA from the parent peptides, suggesting a new/combined mechanism of action. The second hybrid peptide showed similar PCA values to the parent peptide. Here a cysteine bridge that will be cleaved inside the cells sets both parents peptides free. The most active peptide of the both induces the most changes and therefore the signal is closer to that parent peptide. In this experiment, BioSAXS was able to contribute to the elucidation of the mechanism of action and shows the potential to identify the best candidates for further investigation. The results were confirmed by TEM.

### A model for changes observed by BioSAXS to major structural components of *E. coli*

*E.coli* cells were treated with three antibiotics (tetracycline, chloramphenicol and rifampicin) for 4 h and compared with untreated bacteria. The BioSAXS experiments were performed at the P12 BioSAXS beamline at PETRA III (EMBL/DESY) in Hamburg, Germany. In addition, ultra-small-angle X-ray scattering (USAXS) experiments were performed at the USAXS instrument at beamline 15ID (now located at the 9ID) at the Advanced Photon Source (APS), Argonne National Laboratory, in Argonne, USA. The data was analyzed using a model that constructs the bacteria in a hierarchical way consisting of a set of more or less globular entities. The scattering form factor was used to model the SAXS curve (Glatter and Kratky, 1982). The internal structure was modelled as set of spheres of different sizes. Three different populations had to be assumed to consider the shoulders in the SAXS curve and each population corresponds to a certain size. The results from the fit could be assigned to three prominent intracellular components, DNA, proteins and ribosomes. The smallest size with 3.4 nm was assigned to proteins. The second population with 10 nm was assigned to DNA fibrils. The third population with 24 nm was assigned to ribosomes. After treatment with chloramphenicol and tetracycline, no change in protein size was observed, and the volume occupied by protein remained constant. In both cases, the volume occupied by ribosomes was reduced while the radius stayed constant. After treatment with tetracycline DNA radius increased while the occupied volume was reduced. Chloramphenicol had a similar impact on DNA like tetracycline. After treatment with rifampicin, the radius and volume of proteins and ribosomes stayed constant. The radius of DNA increased also the volume occupied by DNA increased. The analysis stresses that indeed the shape change in the SAXS curves relates to intracellular reorganizations of prominent structural units within the bacteria due to the antimicrobial treatment and the reaction of the bacteria to the assault. Such reorganizations are specific responses to the mode of action of the antimicrobial compounds.

### Expanding to gram-positive bacteria and yeast

The first applications of the BioSAXS method were conducted on the gram-negative bacteria *E. coli*. The next step was to test whether the method could be used for gram-positive bacteria as well. There are immense architectural and structural differences between the two types of bacteria. Indeed, the measured SAXS curves clearly showed different shapes for the two bacterial classes, see Figure 2D. PCA of the two curves results in a strong separation on the opposite side of the graph, see Figure 2E. Two newly developed antimicrobial peptides (AMPs) that were discovered by SPOT synthesis screening were investigated (Ashby et al., 2017; López-Pérez et al., 2017). AMPs are a new class of antibiotics that have potential as novel antimicrobial drugs with different modes of action compared to conventional antibiotics (Ashby et al., 2014; Czaplewski et al., 2016). In this BioSAXS study, the peptides were tested on gram-negative *E. coli* and on gram-positive methicillin-resistant *Staphylococcus aureus* (MRSA). Whereas the SAXS curves of the untreated bacteria look very different, after treatment with AMPs for 10 and 40 min, the shape of the curves became similar to each other, see Figure 2D. PCA was used to determine the differences in the SAXS curves (Von Gundlach et al., 2016a). As expected, PCA showed different values for non-treated bacteria. After peptide treatment, PCA showed comparable results for both bacterial species, see Figure 2E. This is an indication of a similar structural change within the bacteria, suggesting a similar mechanism of action in both *E. coli* and MRSA. In this work, BioSAXS could demonstrate the ability to differentiate between gram-positive and gram-negative species for the first time. Especially for spherical bacteria, BioSAXS offers an alternative as the structural changes are difficult to detect in TEM.

BioSAXS was used to study ultrastructural features in vancomycin susceptible *Staphylococcus aureus* (VSSA), heterogeneous vancomycin-intermediate *S. aureus* (hVISA) and vancomycin-intermediate *S. aureus* (VISA) cells. In the untreated strains, the VISA cells showed different SAXS patterns from those of the VSSA and the hVISA, whereas the vancomycin treated cells of hVISA and VISA displayed similar SAXS patterns. Analysing the SAXS curves more in detail, it was detected that under the influence of vancomycin the ribosome of VSSA was significantly smaller than that of hVISA. Without vancomycin treatment, there was no statistically significant difference. BioSAXS can thus deliver insights on bacterial adaptation under vancomycin-mediated stress (Wongthong et al., 2021).

In preliminary experiments, it was shown that five antimicrobial peptides and flucocytocin had induced different ultrastructural changes in the yeast *Candida albicans*. All peptides showed different scattering curves compared to untreated cells or treated with the common antifungal flucytosine. Two of the peptides showed similar activity. Despite the more complex structure of the eukaryotic cells, it was possible to measure and compare the scattering curves of antifungal drugs, making BioSAXS also available for the search of novel antifungals.

## Discussion

BioSAXS is emerging as a novel tool in the antimicrobial drug discovery pipeline. Its strengths are fast measuring time (1–2 s) while simultaneously averaging across millions of cells providing excellent statistics, applicable for gram-positive, gram-negative and yeast cells. The intracellular responses can readily be represented in two-dimensional graphs using a principal component analysis. Among the few weaknesses of the method is the small number of BioSAXS beamline endstations at synchrotrons and the inherently limited beamtime. While the method is not meant to substitute established antimicrobial screening assays or methods to elucidate the precise mode of action, it adds an important information to whether new mode(s) of action in the group of novel antimicrobials are present or not. This contribution can accelerate, enrich and de-risk the screening and ultimately the development of novel antimicrobial drugs. In the future, synchrotron facilities with higher intensity will ensure that even more samples can be measured in the same time. This will provide even higher statistics, which also facilitate the classification. Also the development of more powerful laboratory sources increases the applicability of this method. New detection methods based on machine learning will allow better classification of the substances.
